# The correlation between clinical and radiological severity of osteoarthritis of the knee

**DOI:** 10.1051/sicotj/2022014

**Published:** 2022-04-06

**Authors:** Wynand Steenkamp, Pududu Archie Rachuene, Roopam Dey, Nkosiphendule Lindani Mzayiya, Brian Emmanuel Ramasuvha

**Affiliations:** 1 Sefako Makgatho Health Sciences University Molotlegi St., Ga-Rankuwa Zone 1 Ga-Rankuwa 0208 South Africa; 2 Department of Human Biology, Department of Surgery, Faculty of Health Sciences, University of Cape Town Cape Town 7935 South Africa

**Keywords:** Osteoarthritis, Knee, Functional impairment, Radiologic grading

## Abstract

*Introduction*: Primary osteoarthritis (OA) is a common cause of knee pain. Appropriate management of knee OA is based on clinical and radiological findings. Pain, deformity, and functional impairments are major clinical factors considered along with radiological findings when making management decisions. Differences in management strategies might exist due to clinical and radiological factors. This study aims at finding possible associations between clinical and radiological observations. *Methods*: A prospective cross-sectional study of 52 patients with primary osteoarthritis of the knee managed conservatively at a tertiary hospital arthroplasty clinic was conducted for three months. English speaking patients with primary OA were identified and included in this study. Pain and functional impairment were assessed using Wong-Baker Faces pain scale, The Knee Society Score (KSS), and Western Ontario and McMaster Osteoarthritis Index (WOMAC). The Body Mass Index (BMI) of all participants was measured. Standard two views plain radiographs were used for radiographic grading of the OA. Anonymized radiographs were presented to two senior consultant orthopaedic surgeons who graded the OA using Kellgren and Lawrence (KL) and Ahlbäck classification systems. The severity of the functional impairment and pain score was then compared to the radiological grading. *Results*: The average age of our participants was 63 ± 9 years. Their average BMI was 34.9 ± 8.4 kg/m^2^, median self-reported pain, total WOMAC, and pain WOMAC scores were 8, 60, and 13, respectively. We observed no significant correlation between BMI and pain scores. Inter-rater reliability for KL and Ahlbäck grading was strong. There was no significant correlation between WOMAC scores and the radiological grades. *Conclusion*: There was no correlation between pain and functional scores, patient factors and radiological severity of OA of the knee.

## Introduction

Osteoarthritis (OA) is the most common joint pathology seen worldwide and is the leading cause of disability in the United States [1]. It affects over 40 million people in Europe [2]. Even though the exact cause is still unclear, numerous contributing factors have been identified. The common final pathway is characterized by progressive cartilage matrix degradation to which an ineffectual attempt at repair is made. This leads to cartilage failure causing joint pain, loss of joint function and eventually deformity [3]. Patients commonly present with multiple joint involvements, with the knee being affected in 6% of the adult population [4]. Literature suggests that up to 19% of the rural community in Africa have symptomatic OA of the knee, and a population-based study from a South African rural setting reported a knee osteoarthritis prevalence of 33.1% among adults aged over 35 years [5, 6].

Diagnosis of osteoarthritis of the knee is based on clinical and radiological findings. No universally accepted guidelines or diagnostic criteria h exists [3]. The typical clinical features of OA of the knee include knee pain, decreased range of motion, crepitations, bony tenderness, knee bony enlargement and instability [7]. Knee pain is the most common symptom, and its cause is multifactorial, with both nociceptive as well as neuropathic mechanisms contributing towards it. The cartilage damage, subchondral bone pathology, periosteum, synovium as well as soft tissue have all been thought to contribute to the pain [8, 9].

Grading the clinical severity of knee OA needs to consider multiple factors. Acute pain can be graded using visual analogue or numeric scores. But chronic pain and functional impairment require a more comprehensive grading system. The Knee Society Score (KSS) and Western Ontario and McMaster Osteoarthritis Index (WOMAC) provide a holistic understanding of the impact and severity of the OA in the knee [10, 11].

Despite recent advances in imaging modalities, plain radiographs remain the gold standard imaging modality in diagnosing OA of the knee and ruling out other causes of knee pain [7]. The X-ray views required to assess all three compartments of the knee include weight-bearing anteroposterior and lateral views, Rosenberg view, and the skyline view. Although X-rays can readily be used to detect bony changes secondary to osteoarthritis, the amount of soft tissue involvement remains unclear. Measuring the joint space on X-rays is used as an indirect method to assess the joint cartilage. Unfortunately, the joint space consists of cartilage and includes other soft tissue structures such as the menisci, ligaments, and synovium. Osteophyte formation, joint surface deformation, subchondral sclerosis and cysts make up the typical X-ray features of osteoarthritis [12].

As early as 1957, Kellgren and Lawrence (KL) described a radiographic classification system for osteoarthritis. It considers four features: 1. Joint space narrowing (JSN), 2. osteophyte formation on the joint margins or tibial spines, 3. subchondral sclerosis, and 4. bone-end deformation. Although it has some limitations, it is still the most commonly used grading system [13]. In 1968 Ahlbäck investigated the radiological appearance of the knee in osteoarthrosis, and subsequently published the Ahlbäck classification in 1980 [14, 15]. In contrast to the KL classification, which emphasizes the formation of osteophytes, the Ahlbäck classification focuses more on the amount of joint space narrowing and bone attrition.

A review of the literature showed inconsistent results between the severity of clinical features compared to radiological gradings (Table 1). Szebenyi et al. reported that patients were more likely to have pain if radiological changes were seen in the tibiofemoral compartments and the patellofemoral compartments. They also found that subchondral sclerosis was linked to pain rather than a global grading as by KL [16]. Polat et al. considered that some patients with knee OA had a neuropathic pain component that contributed to the overall joint pain. The authors also found that the radiologic grading correlated to the patients’ age than the reported degree of pain [8]. Contrary to this, Kocak et al. found that patients with KL grades III and IV radiological features had more pain, lower muscle strength, range of motion, and functional scores than patients with KL I and II [17].

**Table 1:** Summary of related published articles and their findings.

Author	Title	Relevant results
Szebenyi et al. [16]	Associations between pain, functional, and radiographic features in osteoarthritis of the knee	• Higher levels of pain reported if all compartments of the knee involved
		• Subchondral sclerosis linked to pain, rather than global radiological grading
Polat et al. [8]	Is there a possible neuropathic pain component in knee osteoarthritis	• Radiological grading severity linked to age rather than degree of pain reported
Kocak et al. [17]	Associations between radiographic changes and function, pain, range of motion, muscle strength and knee function score in patients with osteoarthritis of the knee	• KL grade III and IV correlated to more severe clinical features
Talic-Tanovi et al. [19]	Comparison of clinical and radiological parameters at knee osteoarthritis	• Females had higher levels of OA of knee
		• No significant correlation between clinical and radiological severity of OA of knee
Zheng et al. [21]	Body mass index and risks of knee osteoarthritis: systematic review and meta-analysis of prospective studies	• BMI is an independent predictor of OA of the knee
Alahmari et al. [23]	Mediating role of body mass index in knee osteoarthritis	• Higher BMI levels correlated to more severe levels of pain reported

Knee OA patients in sub-Saharan Africa often belong to the underprivileged sections of society. Often, they are incapable of affording a thorough clinical examination, and the clinician has to decide depending on the only available information, which can either be radiological or clinical. There is no evidence on the association of clinical and radiological observations in the sub-Saharan population Understanding such associations will assist clinicians in managing knee OA patients. Therefore, the primary aim of this study was to compare radiographic findings to pain severity and functional impairments in patients with osteoarthritis of the knee joint. The secondary aim was to assess if Body Mass Index (BMI) contributed to the severity of either clinical or radiological parameters.

## Materials and methods

A prospective cross-sectional study was conducted from April 2021 until June 2021 after obtaining institutional ethics clearance and hospital gatekeeper permission. A total of 52 English speaking patients with primary knee OA who were treated conservatively and followed up at a tertiary academic hospital arthroplasty clinic were included in this study. The average age of the cohort was 63 ± 9 years. Patients with secondary knee arthritis were not included in the study. Prior to participating in this study, participants gave their informed consent. They were then asked to fill out a standard questionnaire with their demographic information (age, sex, weight, and height). The subjects also filled in self-reported pain grading information (numeric pain rating scale and Wong-Baker FACES pain rating scale) and functional assessment information (Knee Society Score and Western Ontario and McMaster Osteoarthritis Index).

As part of our institution’s standard of care for patients with OA of the knee, plain knee X-rays are repeated and reviewed at 3 monthly intervals to evaluate the progression of the disease. The principal author provided reviewers with anonymized X-rays consisting of an anteroposterior and a lateral view. To improve the validity and reliability of this analysis, 2 orthopaedic consultant reviewers were simultaneously tasked to assess the X-rays independent of each other and grade them using standard KL as well as Ahlbäck classification systems.

Data was compiled by the principal investigator and assessed only after all the data had been collected. The functional impairment and pain severity were compared to the radiological grading to determine if any association existed. Secondly, the data was assessed to determine whether there was any correlation between the patients’ age or BMI compared to clinical and radiological severity.

All statistical analyses were performed in IBM SPSS v.27 (IBM Corp., Armonk, NY, USA). Descriptive statistics were presented as average (standard deviation) or median (max; min) depending on the distribution. Inter-rater reliability was calculated using the intra-class correlation coefficient (ICC) with a two-way mixed model absolute agreement. ICC values were presented as (average measures ICC; 95% confidence interval). Kendall’s tau correlation coefficient was calculated between WOMAC scores and radiographic gradings. Correlation coefficients were also calculated between pain scores (self-reported and WOMAC) and patient BMI. The cut-off for statistical significance for all the tests was set as *p* < 0.05.

## Results

**Figure 1:**
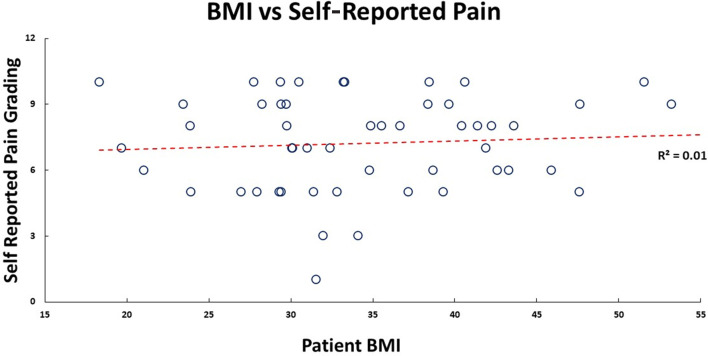
Patient self-reported pain score plotted against their BMI, and their linear relationship is shown by the red dashed trend line.

**Figure 2:**
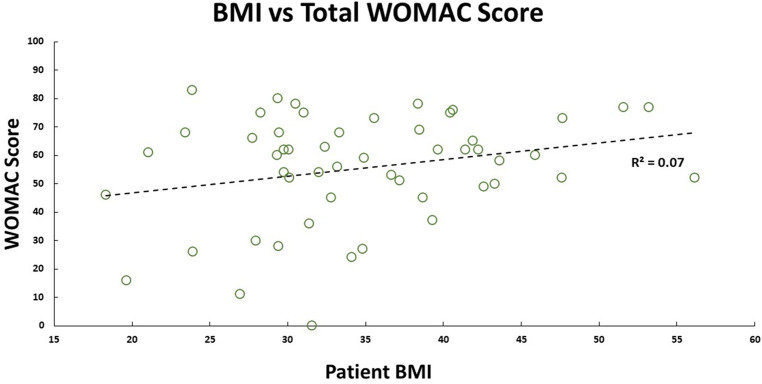
Patient WOMAC score plotted against their BMI, and their linear relationship is shown by the black dashed trend line.

Our cohort had 92% female participants. Their average weight, height, and BMI were 91.8 (±21.3) kg, 1.6 (±0.1) m, and 34.9 (±8.4) kg/m^2^, respectively. There were 69.2% obese participants, 19.2% overweight participants, 11.2% had healthy BMI. The median self-reported pain grading was 8 (1; 10), and Knee Society Score was 20 (0; 40). Medians for WOMAC scores were – total: 60 (0; 83); pain: 13 (0; 17); stiffness: 5 (0; 8); functional: 42 (0; 62). There was no significant correlation between pain (self-reported, KSS, and WOMAC) with participant’s BMI (Figures 1 and 2) and age. No walking aid was used by 50% of our participants; 25% of them used a single crutch, followed by 15.4% who used double crutches. The rest of them used a walking stick (7.7%) and a walker (1.9%).

**Figure 3:**
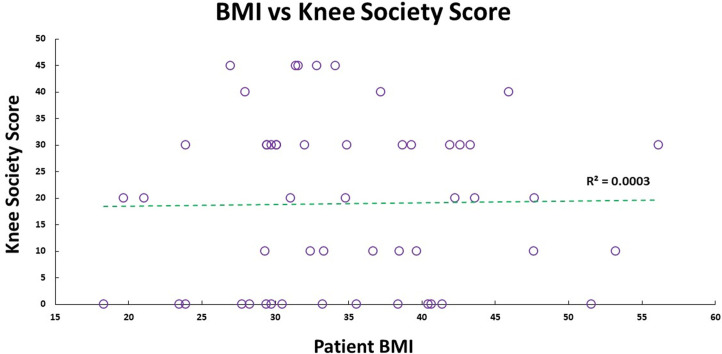
Patient Knee Society score plotted against their BMI, and their linear relationship is shown by the green dashed trend line.

**Figure 4:**
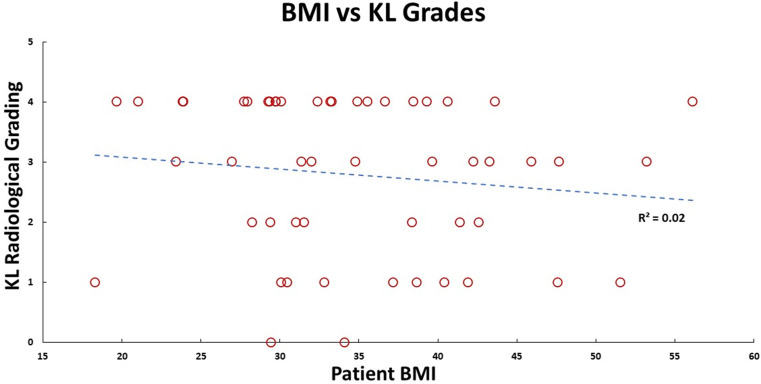
Patient Kellgren and Lawrence radiological grading plotted against their BMI, and their linear relationship is shown by the blue dashed line.

**Figure 5:**
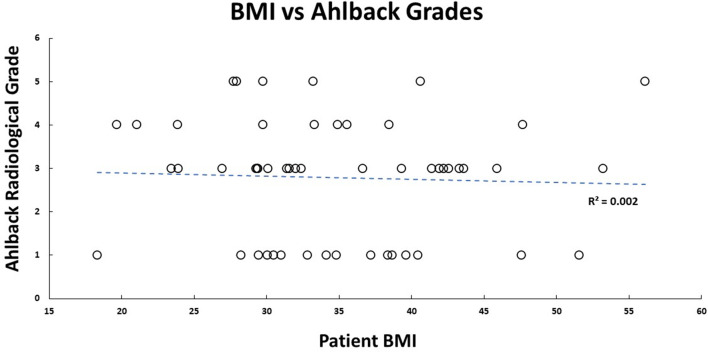
Patient Ahlbäck radiological grading plotted against their BMI, and their linear relationship is shown by the blue dashed line.

**Figure 6:**
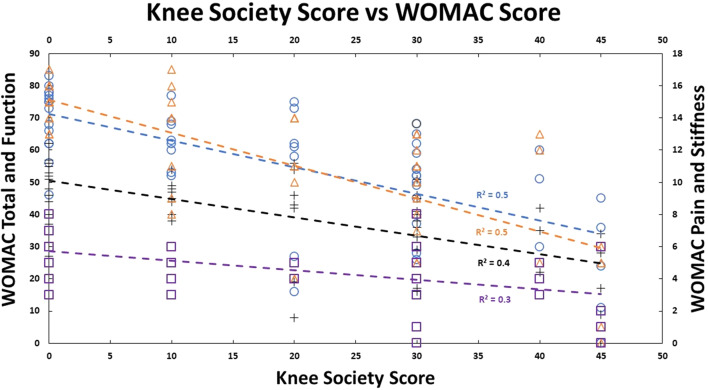
Patient WOMAC score components plotted against their Knee Society Scores (KSS). Total WOMAC score is represented by the blue color, the Pain component is represented by the orange color, the Functional component is represented by the black color, and the Stiffness component is represented by the purple color. Each component’s linear relationship with KSS is represented by the dashed in their respective colors.

There was strong inter-rater reliability (*p* < 0.001) for KL (ICC: 0.82; 95% CI: 0.68–0.89) and Ahlbäck (ICC: 0.87; 95% CI: 0.77–0.92) radiographic classifications. The median KL grade was 3 (0; 4), and Ahlbäck grade was 3 (1; 5). The radiological scores had a statistically significant (*p* < 0.01) medium correlation (0.73) with each other (Figures 3–5). There was no significant correlation between the radiological gradings with WOMAC scores and KSS (Figure 2). The Knee Society Score had a statistically significant (*p* < 0.01) negative correlation with WOMAC scores, ranging from −0.41 to −0.61 (Figure 6).

## Discussion

Deciding on a management plan for patients with OA of the knee can be complex. Treatment needs to be individualized per patient, and the main aim of treatment should be to relieve pain and optimize function [18]. Treatment usually consists of conservative management initially, with surgery as an option for those who do not respond. Both clinical as well as radiological parameters, should be considered when such decisions are made. For this reason, it is essential to understand the relationship between the two.

Our study did, however, have some limitations. Firstly, a single interview was conducted with the participants, and we did not follow them up to assess if the progression of radiological severity affected their clinical severity. Secondly, the study was conducted at a tertiary hospital. Therefore, these patients had already failed treatment at primary or secondary care. This might influence the severity of symptoms reported by participants.

**Figure 7:**
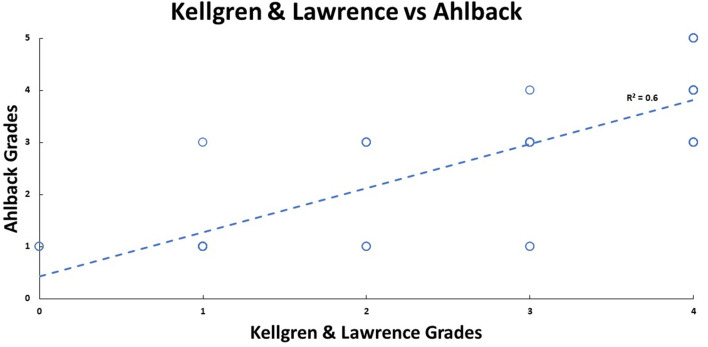
Patient Ahlbäck radiological grades plotted against Kellgren and Lawrence’s radiological grades, and their linear relationship is shown by the blue dashed line.

In our study, the vast majority (92%) of participants were female. This correlates to previous studies that showed a higher incidence of OA in females [14, 19]. Western Ontario and McMaster Osteoarthritis Index (WOMAC) score consist of three subsections: Pain, stiffness and function. It provides a global picture of the symptoms and their impact caused by OA of the knee. The KSS is a functional assessment of the impact of pain caused by OA of the knee. Our study found no correlation between either the WOMAC score or KSS score when compared to the severity of the radiographs as classified by either KL or Ahlbäck (Figure 7). This is in keeping with the results found by Talic-Tanovi et al. and Szebenyi et al. However, we did not, find a correlation between subchondral sclerosis and pain as found by Szebenyi et al. [8, 16, 19]. This is in contrast to Kocak et al., who found that higher patients with higher-grade radiological gradings suffer from more severe clinical features [17].

This could be that the exact origin of the pain and sequelae thereof is still poorly understood. However, it suggests that OA is not just purely a degenerative condition of the cartilage but does involve an inflammatory as well as soft tissue component. This supports the study by Roemer et al. that suggested that OA is not just a disease of the cartilage but involves the whole joint as well as other soft tissue that eventually leads to joint failure [20].

A systematic review and meta-analysis by Zheng et al. showed that body mass index (BMI) was an independent predictor for OA of the knee [21]. It has also been shown that obesity is associated with both incidences as well as the progression of OA [22]. The body mass index of participants in our study ranged between 26.5 and 43.3, with a median score of 34.9. No correlation between the BMI and clinical or radiological severity was observed. This contrasts with previous studies that showed that a higher BMI correlates with more severe pain in patients with OA of the knee [23]. The reason for this could be that our median BMI was 34.9, thus the majority of our patients being obese or overweight already.

## Conclusion

The discordance between clinical and radiological features of OA of the knee found in our study is in keeping with multiple previous studies. A thorough clinical evaluation of these patients is essential to determine the severity of the condition and decide on the appropriate management. Further studies are needed to identify the exact origin of the pain. Although X-rays still form part of the complete workup of patients with suspected OA of the knee, one needs to consider its shortcomings to grade the severity. Measuring the joint space as an indirect indicator of the cartilage quality is an easy and readily available technique, but it cannot be used in isolation to determine management. The gold standard imaging modality is yet to be determined, and further studies are needed.

## Conflict of interest

W. Steenkamp: The author declares that they have no relevant financial or non-financial interests to report.

PA Rachuene: The author declares that they have no relevant financial or non-financial interests to report.

R Dey: The author declares that they have no relevant financial or non-financial interests to report.

NL Mzayiya: The author declares that they have no relevant financial or non-financial interests to report.

BE Ramasuvha: The author declares that they have no relevant financial or non-financial interests to report.

## Funding

This research did not receive any specific funding.

## Ethical approval

This study received ethical approval from the Ethics committee of Sefako Makgatho University under the protocol number SMUREC/M/51/2021: PG.

## Informed consent

Written informed consent was obtained from all patients as well as the surgeons who reviewed X-rays.

## Authors contributions

W. Steenkamp: Conceptualization, manuscript preparation and critical manuscript review.

PA Rachuene: Conceptualization, manuscript preparation and critical manuscript review

R Dey: Data analysis, manuscript preparation, critical manuscript review.

NL Mzayiya: Manuscript preparation and critical manuscript review.

BE Ramasuvha: Manuscript preparation, critical manuscript review and senior guidance.
